# Supplementary material for: A Candidate Gene Association Study for Economically Important Traits in Czech Dairy Goat Breeds
Source: Animals (Basel). 2021 Jun 16;11(6):1796. doi: 10.3390/ani11061796 (PMC8234603; doi:10.3390/ani11061796)
Supplement: Supplementary file 1 [file animals-11-01796-s001.zip › animals-1224782-supplementary.pdf]

animals

Article

A Candidate Gene Association Study for Economically Important Traits in Czech Dairy Goat Breeds

Michaela Brzáková [1,*], Jana Rychtářová [2], Jindřich Čítek [3,4] and Zuzana Sztankóová [1]

1 Department of Genetics and Breeding of Farm Animals, Institute of Animal Science, 104 00 Prague, Czech Republic; sztankoova.zuzana@vuzv.cz
2 Department of Biology of Reproduction, Institute of Animal Science, 104 00 Prague, Czech Republic; rychtarova.jana@vuzv.cz
3 Department of Genetics and Agricultural Biotechnologies, Faculty of Agriculture, University of South Bohemia, 370 05 Ceske Budejovice, Czech Republic; citek@zf.jcu.cz
4 Department of Infectious Diseases and Preventive Medicine, Veterinary Research Institute, 621 00 Brno, Czech Republic
* Correspondence: brzakova.michaela@vuzv.cz; Tel.: +420-606-794059

Supplementary Material

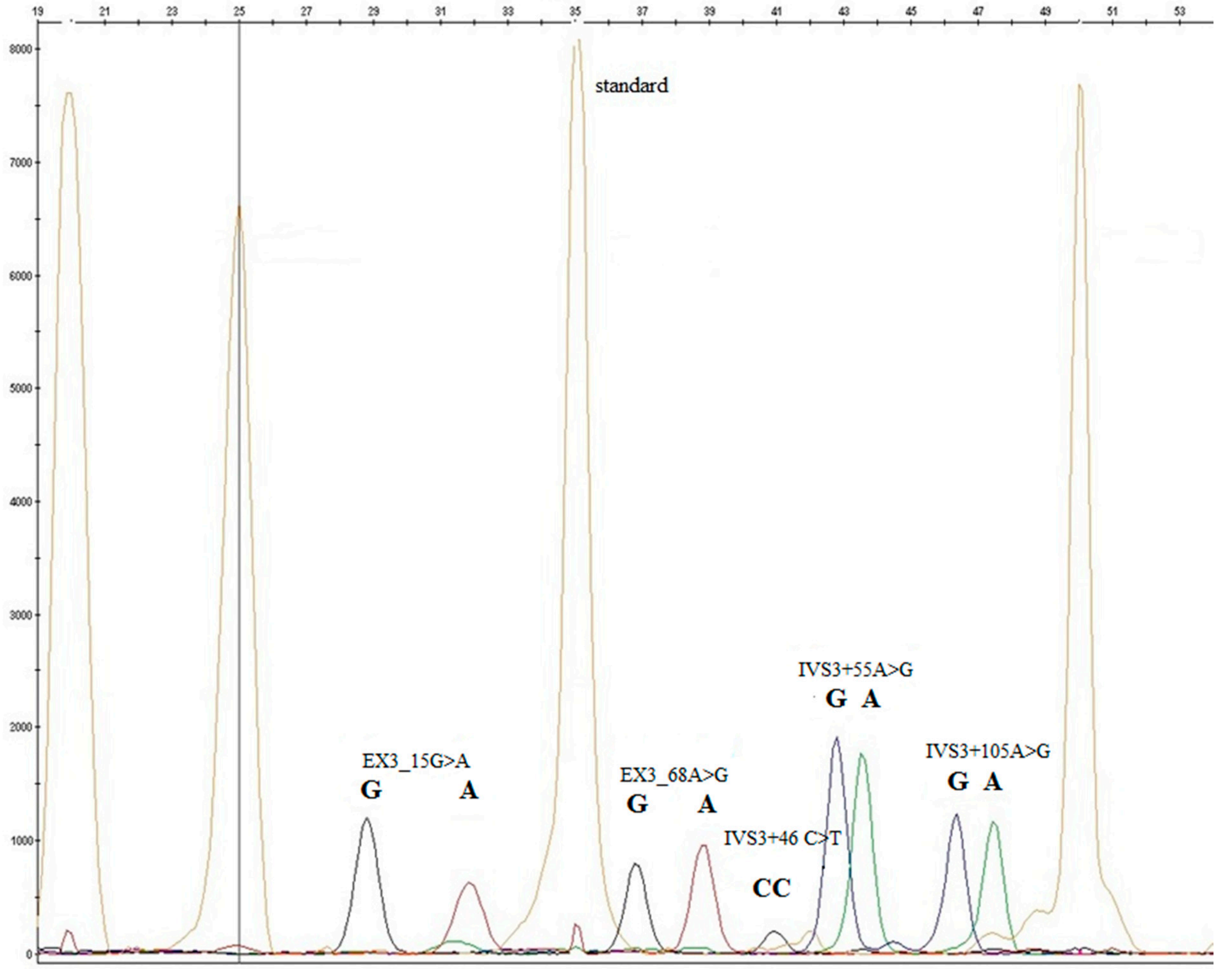

A

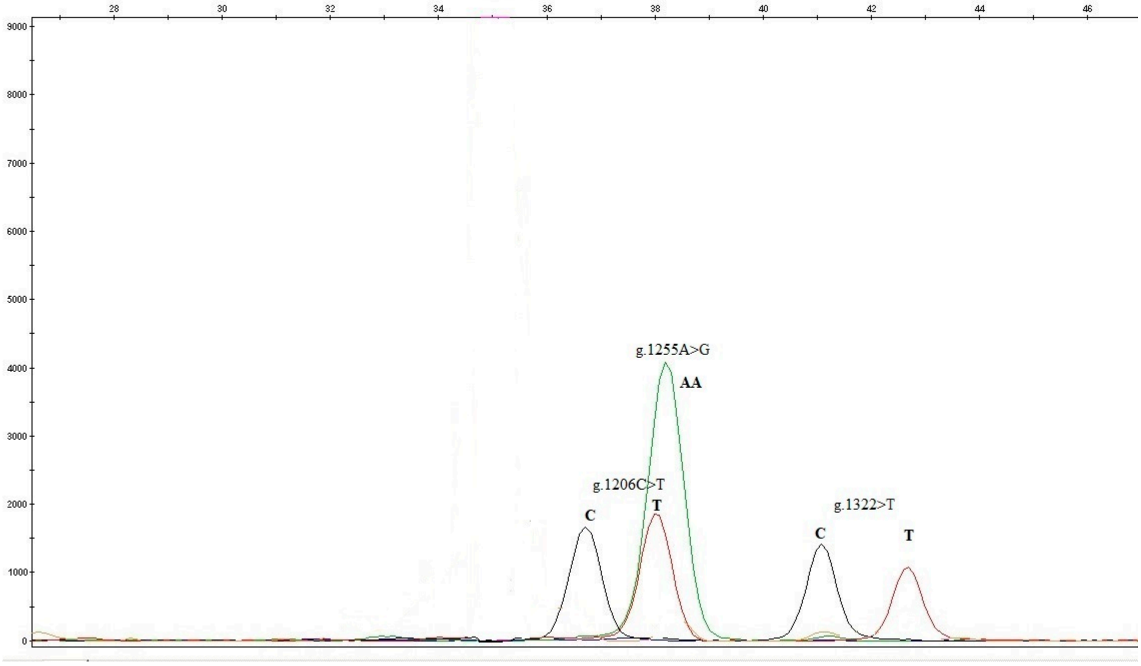

B

Figure S1. Electropherograms. (**A**) Electropherogram of SNPs (AH011188.2; AF422168.1) in the Stearoyl-coenzyme A desaturase (SCD) gene analysed with Gene Mapper software. (**B**) Electropherogram of SNPs (AJ292286) in the Acetyl-coenzyme A carboxylase α (ACACA) gene analysed with Gene Mapper software.

Table S1. Primers used for the amplification PCR product and extension primers (PEA analysis) of the goat Stearoyl-CoA desaturase (GenBank AH011188.2; AF422168.1), Acetyl-CoA carboxylase (AJ292286) cDNA, and annealing temperature (Tm)[1].

Locus	Primer name and sequence (5´--3´)	Tm °C
ACACA	F: gggCCCCTTTACgTTTCTgT	59
	R: AgCTTCTgCCTTAgCTgCA	
PEA	PIII[a] K-ACACA (1206): $(AT)_4$ATTTCCCTCTTgACCTgCTCT	
	K-ACACA (1255): gTCTTgTTgTgATTgggTCTCAg	
	K-ACACA (1320): $(AT)_7$ATTTTCTgggCATAgCTgTCC	
SCD	F: TCCTAAgCTTATTCCAgCCCC	59.5
	R: gCCAgTCACTCAgAAgTACCC	
PEA	Exon3 K – EX3_15G>A: TgCCCAgggCACTCATCA	
	Exon3 K – EX3_68A>G: $(AT)_3$gCAgCCgAgCTTTgTAggT	
	Intron3 K – IVS3+46C>T: $(AT)_4$AgCTCTTTTgCTCCTCACTCTTTA T	
	Intron3 K – IVS3+55A>G: $(AT)_6$ ATCTCCTCACTCTTTATCgATgAgCC	
	Intron3 K – IVS3+105A>G: $(AT)_{11}$ AGAgggACAgCACCTggATA	

ACACA - Acetyl-CoA carboxylase, SCD - Stearoyl-CoA desaturase, PIII[a] = promotor III – 5´UTR.

Table S2. Thermal cycling conditions of Stearoyl-CoA desaturase (SCD) and Acetyl-CoA carboxylase α (ACACA) loci.

SCD		ACACA
95 °C for 4 min		95 °C for 2 min
94 °C for 30 sec		95 °C for 30 sec
59,5 °C for 30 sec	29 cycles	59 °C for 45 sec
72 °C for 40 sec		72 °C for 1 min
72 °C for 10 min.		72 °C for 5 min
